# Light-driven dynamics: unravelling thiol-redox networks in plants through proteomics

**DOI:** 10.1093/plphys/kiae076

**Published:** 2024-02-12

**Authors:** Gustaf E Degen

**Affiliations:** Assistant Features Editor, Plant Physiology, American Society of Plant Biologists; Plants, Photosynthesis and Soil, School of Biosciences, University of Sheffield, Firth Court, Western Bank, Sheffield S10 2TN, UK

Unlike animals, plants cannot hunt for prey or seek shelter; instead, they use photosynthesis by harvesting the energy from sunlight to fix atmospheric carbon dioxide into biomass, allowing them to grow and spread. Owing to their sessile nature, plants have evolved ingenious mechanisms to protect themselves from too much sunlight and to adapt their metabolism to fluctuating environmental conditions. One of the mechanisms is the thiol-based redox regulation of protein activities, an ancient and evolutionary conserved mechanism of post-translational modification. Thiol groups of cysteine residues in proteins form disulfide bonds when oxidized, but this bond is broken into two thiol groups upon reduction by a group of small proteins called thioredoxins (Trxs) (Fig. 1A).

**Figure 1.**
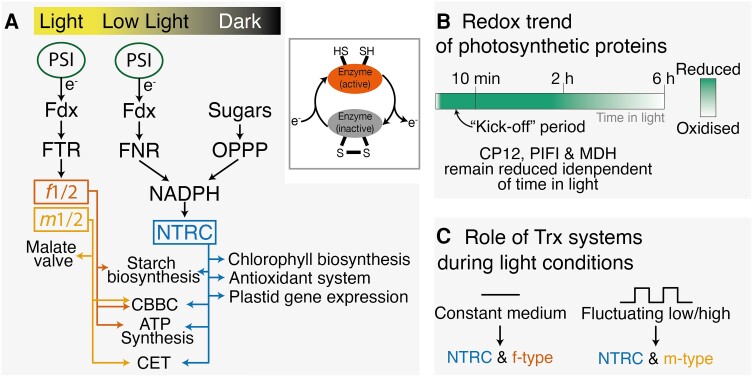
**A)** An overview of the two Trx systems in plants. The Fdx-dependent system uses reduced ferredoxin, whereas the NTRC system uses NADPH from FNR or the oxidative pentose phosphate pathway (OPPP). In the target enzymes, the disulfide bond is reduced via one of the thioredoxin systems and thus activated. Reoxidation of the thiols returns the enzyme into its inactive state. **B)** Oxidation trend of photosynthetic proteins. During the first 10 min in the light, the photosynthetic proteins are rapidly reduced and slowly re-oxidize in the light, except for CP12, PIFI, and MDH. **C)** The NTRC and *f*-type thioredoxins play a role during constant light, whereas the NTRC and *m*-type thioredoxins play a role during fluctuating light. Abbreviations: PSI, Photosystem I; Fdx, reduced ferredoxin; FTR, ferredoxin-thioredoxin reductase; FNR, ferredoxin-NADP oxidoreductase; CBBC, Calvin-Benson-Bassham cycle; CET, cyclic electron transfer; OPPP, oxidative pentose phosphate pathway; NADPH, nicotinamide adenine dinucleotide phosphate; CP12, Calvin-cycle protein 12; PIFI, POSTILLUMINATION CHLOROPHYLL FLUORESCENCE INCREASE; MDH, NADP-dependent malate dehydrogenase. The Trx-systems overview in (A) was adapted from Geigenberger et al. (2017) and Nikkanen et al. (2019).

In plant chloroplasts, light energy is used to excite electrons, which are transferred along the electron transfer chain in the thylakoid membrane. In the final step, ferredoxin (Fdx) transfers electrons to ferredoxin-NADP oxidoreductase (FNR), which catalyzes the synthesis of NADPH. To effectively respond to variable light environments, plants have evolved two Trx systems. The Fdx-dependent system involves the Fdx-dependent Trx reductase (FTR), which uses reduced ferredoxin (Fdx) as an electron source (reviewed in Buchanan et al. 2016). This Fdx-Trx system contains many different types; most important for photosynthesis are the *f-* and *m-*types (Fig. 1A), and they are most active under moderate and high light (Thormählen et al. 2015; Geigenberger et al. 2017; Nikkanen et al. 2019). Because the Fdx-Trx system uses ferredoxin that is reduced in the light, the NADPH-dependent Trx reductase (NTRC) system is required to reduce and activate chloroplast proteins during dark-to-light transitions (Serrato et al. 2004). NTRC can use electrons from photosynthetically synthesized NADPH but can also use NADPH from the oxidative pentose phosphate pathway in the dark. Hence, the NTRC system is active in the dark or during low light (Fig. 1A), so that during dark-to-light transitions, photosynthetic proteins can be activated and photosynthesis can be “kickstarted.” Redox regulation via the two Trx systems allows for balancing of light and carbon-fixation reactions. For example, the chloroplast NADH dehydrogenase-like complex (NDH) participates in one of the two cyclic electron transfer (CET) pathways and is activated under low light by NTRC. However, under higher light, TRX*m* (an Fdx-depdentent Trx) inhibits the NDH complex and activates the PROTON GRADIENT REGULATION 5-depdentent CET pathway and proteins in the CO_2_-fixing Calvin-Benson-Bassham cycle (CBBC). This illustrates how these two Trx systems ensure proper tuning of light and carbon reactions in response to the light environment. Even though we have a good understanding of the Trx systems, a quantitative investigation into the thiol-disulfide proteome in response to different light treatments is lacking.

In this issue of *Plant Physiology*, (Hou et al. 2024) use a biotin-switch assay to quantify the redox changes of proteins in response to photoperiod and light intensity. In this assay, proteins were extracted from *Arabidopsis thaliana* leaves in the presence of N-ethylmaleimide, which reacts with free thiol residues in proteins to form a strong C-S bond, irreversibly blocking free thiols. Then, the disulfide bonds in proteins were reduced with dithiothreitol, and the released free thiol groups were subsequently labeled with biotin, allowing affinity purification of proteins with disulfide bonds using a Streptavidin column. The method only identifies previously oxidized proteins. To verify their biotin approach, the redox status of some CBBC proteins were analyzed using Western blot. This highlights the power and precision of proteomics and shows that it can be used as an alternative to Western blots. Finally, the purified proteins were identified with mass spectrometry and expressed as log_2_-fold change relative to proteins at the end of the night; a +1.5-fold change meant that proteins were more oxidized, whereas a −1.5-fold change meant that proteins were more reduced.

In the first experiment, leaf materials were harvested in the dark and 10, 120, and 360 min into the light period. This experiment revealed that mainly proteins located in the plastid with photosynthetic function showed changes in redox status. The vast majority of photosynthetic proteins were in a reduced state after the first 10 min. Interestingly, the CBBC-regulator CP12, which regulates the formation of a complex between glyceraldehyde-3-phosphate dehydrogenase (GAPDH) and phosphoribulokinase (PRK) in response to changes in light intensity, remained reduced during the entire light period, preventing the formation of the inhibitory complex and keeping the CBBC active. Curiously, Rubisco activase, which activates the CO_2_-fixing enzyme Rubisco, showed progressive oxidation during the experiment, suggesting that these two CBBC-regulators have different regulatory roles. Overall, this experiment showed that the first 10 min of illumination are crucial to “jump-start” the photosynthetic processes (Fig. 1B).

To investigate the roles the TRX system plays in regulating the thiol-redox proteome, Hou et al. used mutants deficient in the Fd-TRX system (*trxf1f2* and *trxm1m2*) as well as the *ntrc* mutant. In this experiment, plants were exposed to either constant medium light or light fluctuating between low and high light intensities. They found that during constant light, the *f*-type Trxs and NTRC play the dominant role. However, during fluctuating light *m*-type Trxs and NTRC are more important.

Improving crop yields by optimizing photosynthesis has become a hot topic in recent years (Ort et al. 2015). One of the bottlenecks of photosynthesis appears to be the suboptimal response to fluctuating light, making this a promising target for engineering crops with improved photosynthetic capacity (Wang et al. 2020). The insights from Hou et al. published in this issue of *Plant Physiology* add to the growing body of work on the role of the TRX system during fluctuating light levels. For example, overexpression of NTRC in Arabidopsis raised carbon fixation by about 20% (Nikkanen et al. 2016), although protein amount and subcellular localization are important to achieve this effect. Nonetheless, the TRX system is certainly a promising target for optimizing light harvesting and carbon fixation during photosynthetic induction and fluctuating light.

## Dive Curated Terms

The following phenotypic, genotypic, and functional terms are of significance to the work described in this paper:

NADPH CHEBI: CHEBI:16474

PIFI Gramene: AT3G15840
PIFI Araport: AT3G15840

NADH CHEBI: CHEBI:16908

thiol group CHEBI: CHEBI:29917

MDH Gramene: AT3G47520
MDH Araport: AT3G47520
